# National Medical Convention

**Published:** 1847-06

**Authors:** 


ARTICLE IV.

### NATIONAL MEDICAL CONVENTION.

The meeting of this association which met in Philadelphia on the fifth of May last, was large, and composed of many of the first men of the nation. All the States of the Union were represented except Maine, Alabama, Arkansas, Wisconsin, Texas, and Floridas.

There were seventy-two medical associations represented. Some of them by a number of delegates, whose credentials were examinad by a committee and they took their seats.

Dr. Holmes, of Massachusetts, from a committee of one from each State represented, appointed for the purpose, reported the following named members for officers of the convention, who were unanimously appointed, viz.:

Dr. J. Knight, of Connecticut, *President*.

Drs. Alex. H. Stevens, of New York, G. B. Wood, of Philadelphia, A. H. Buchanan, of Tennessee, and John Harrison, of Louisiana, *Vice Presidents*.

Drs. R. D. Arnold, of Georgia, A. Stille, of Philadelphia, F. C. Stewart, of New York, *Secretaries*.

The following account of the first day's proceedings we take from the New York Med. and Surg. Reporter:

Several propositions were made to admit gentlemen of the medical profession, not delegates to the Convention, to seats in the body, all of which were voted down.

The convention then proceeded to the consideration of the

Report on the subject, (which was referred to a Committee at the last meeting of the Convention in 1846,) of instituting a National Medical Association, for the protection of the Medical Profession, &c. The name of the Association to be the American Medical Association.

On motion of Dr. Watson, of New York, the report was laid on the table, and the same ordered to be printed.

The Convention next took up the Report, accompanied by the address of the committee of the last Convention appointed to consider the expediency, &c., if deemed expedient, of the mode of recommending and urging upon the several State governments, the adoption of measures for a registration of the births, marriages, deaths of their several populations.

The report was accepted, and the address adopted and ordered to be printed.

The convention adjourned until 7 P. M.

Evening Session.—The convention re-assembled at seven o'clock, pursuant to adjournment, when

The President laid before the convention a letter from the managers of the House of Refuge, and also one from those of the Pennsylvania Institution of the Deaf and Dumb, inviting the delegates of the national medical Convention to visit those public institutions.

The convention again proceeded to the consideration of reports—the first being that of the committee appointed at the last session on the following resolution, viz.:

*Resolved*, That it is desirable that a uniform and elevated standard of requirements for the degree of M. D., should be adopted by all the medical schools in the United States, and that a committee of seven be appointed to report on this subject at a meeting to be held in Philadelphia on the first Wednesday in May, 1847.

Dr. Haxall, of Virginia, the chairman, read an elaborate, elegantly written, and argumentative report, of which the above resolution formed the basis. The following important resolutions were embodied in the report, for the consideration and action of the convention.

*Resolved*, 1st. That it be recommended to all the colleges to extend the period employed in lecturing from four to six months.

2d. That no student shall become a candidate for the degree of M. D., unless he shall have devoted three entire years to the study of medicine, including the time allotted to attendance upon the lectures.

3d. That the candidate shall have attended two full courses of lectures, that he shall be 21 years of age, and in all cases shall produce the certificate of his preceptor, to prove when he commenced his studies.

4th. That the certificate of no preceptor shall be received who is avowedly and notoriously an irregular practitioner, whether he shall possess the degree of M. D. or not.

5th. That the several branches of medical education already named in the body of this report be taught in all the colleges; that no less than one hundred lectures be delivered by each Professor, and that the number of Professors be increased to seven.

6th. That it be required of candidates that they shall have steadily devoted three months to dissections.

7th. That it is incumbent upon Preceptors to avail themselves of every opportunity to impart clinical instruction to their pupils; and upon Professors to connect themselves with hospitals, whenever it can be accomplished, for the advancement of the same end.

8th. That it is incumbent upon all schools and colleges granting diplomas fully to carry out the above requisitions.

9th. That it be considered the duty of Preceptors to advise their students to attend only such institutions as shall rigidly adhere to the recommendations herein contained.

The Report was accepted and ordered to be printed.

Dr. James Cowper, from the Committee on the following resolution, passed by the last Convention, made a report thereon, which was received, read and ordered to be printed.

*Resolved*, That it is desirable that young men, before being received as students of medicine, should have acquired a suitable preliminary education, and that a committee of seven be appointed to report on the standard of acquirements which should be exacted of such young men, and to report at a meeting to be held at Philadelphia on the first Wednesday in May, 1847.

The Report of the committee concludes with the following language:

The object to which the Committee has directed its labors, it is believed, can be best effected by the Convention in the following way:

1st. By establishing a uniform standard of preliminary education for medical students, which shall be of a moderate character—in the first instance, too low rather than too high,—and yet of such extent as will insure both the knowledge and the mental discipline necessary to those who would enter a profession full of labor and responsibility, without excluding meritorious young men of limited means and opportunities.

2d. By earnestly recommending every medical preceptor to exact this standard of every young man, before admitting him into his office; and having exacted it, to grant him a written certificate to that effect, specifying also the period of his admission into the preceptor's office, as a proper warrant and credential for the student, when about entering a medical college.

3d. By requesting all the medical colleges of the country to require such a certificate of every student applying for matriculation, and, in publishing their annual lists of graduates, to accompany the name of the graduate with the name and residence of his preceptor, the name of the latter being clearly and distinctly presented, as certifying to the qualification of preliminary education.

These ideas the committee have put into the form of distinct resolutions, which they append to their report, submitting both for the consideration of the convention, and, if it think proper, its adoption.

*Resolved*, That this convention earnestly recommends to members of the medical profession, throughout the United States, to satisfy themselves, either by personal inquiry or the written certificate of competent persons, before receiving young men in their offices, as students, that they are of good moral character, and that they have acquired a good English education, a knowledge of natural philosophy, and the elementary mathematical sciences, including geometry and algebra, and such an acquaintance, at least, with the Latin and Greek languages, as will enable them to appreciate the technical language of medicine, and read and write prescriptions.

*Resolved*, That this convention also recommends to the members of the medical profession of the United States, when they have satisfied themselves that a young man possesses the qualifications specified in the preceding resolution, to give him a written certificate, stating that fact, and recording also the date of his admission as a medical student, to be carried with him as a warrant for his reception into the medical college in which he may intend to complete his studies.

*Resolved*, That all the medical colleges in the United States be, and they are hereby recommended and requested to require such a certificate of every student of medicine applying for matriculation; and, when publishing their annual list of graduates, to accompany the name of the graduate with the name and residence of his preceptor, the name of the latter being clearly and distinctly presented, as certifying to the qualification of preliminary education.

Dr. Bell, of Philadelphia, from the committee on the following resolution, adopted by the convention in 1846, made a very long report thereon, which was accepted, read, and laid on the table.

*Resolved*, That it is expedient that the medical profession in the United States should be governed by the same code of medical ethics, and that a committee of seven be appointed to report a code for that purpose at a meeting to be held in Philadelphia on the 1st Wednesday of May, 1847.

The convention adjourned at half past 9 o'clock, to meet again at 9 o'clock, Thursday morning.

May 6th.—The convention met pursuant to adjournment.

The report of the committee on Medical Education was taken up, on motion of Dr. Hays, and the resolutions severally adopted without amendment, except the seventh, for which, on motion of Dr. W. L. Atlee, the following was substituted:

*Resolved*, That it is incumbent upon the preceptors to avail themselves of every opportunity to impart clinical instruction to their pupils; and upon medical colleges to require candidates for graduation to show that they have attended upon hospital practice for one session whenever it can be accomplished.

The following additional resolution, offered by Dr. Stewart of New York, was adopted:

*Resolved*, That it be suggested to the faculties of the various medical institutions of the country, to adopt some efficient means of ascertaining that their students are actually in attendance upon their lectures.

After receiving an invitation from Dr. Kirkbride to visit the Pennsylvania Hospital for the Insane, under his care, the convention adjourned until nine o'clock to-morrow morning.

May 7th.—Met pursuant to adjournment.

Dr. Haxhall moved to reconsider the vote taken on the fifth resolution adopted yesterday in reference to medical colleges, in which each professor was required to give one hundred lectures to make his course complete in the estimation of the convention; which was carried. The number of lectures was then stricken out, and the resolution adopted.

On motion of Dr. Watson of New York,

The convention took from the table the report of the committee in reference to organizing a National Medical Association, and after considering the articles of constitution some time, they were adopted as a whole.

The convention then took up the consideration of the report of the committee on medical ethics;

Which report was adopted without amendment.

The report of the committee on the subject of separating the business of teaching and licensing was taken up, and, on motion of Dr. Leonard, of Maryland, the following resolution was adopted:

*Resolved*, That in view of the necessity hereby declared for reform in the manner of conferring degrees, the two reports submitted by the committee on the separation of teaching and licensing, be published and referred to the committee on medical education, with instructions to report at the next annual meeting of the American Medical Association.

The convention then adjourned to five o'clock, P. M.

Evening Session.—Dr. Flint of New York, offered the following resolution, which was adopted:

*Resolved*, That it be recommended by this convention to the medical profession of the States, wherein laws sanctioning and providing for the prosecution of dissections do not already exist, to unite in endeavoring to procure the passage of such laws.

Dr. Stewart of New York offered the following:

*Resolved*, That all unfinished business be referred to the "American Medical Association" about to be organized.

*Resolved*, That this convention do now resolve itself into the "American Medical Association," and that the officers of the convention continue to act as officers of the Association until others be appointed;

Which were unanimously adopted.

A committee of one from each State represented was appointed to nominate suitable persons as officers of the association, who reported—

*For President*—Dr. N. Chapman, of Philadelphia.

*For Vice Presidents*—Drs. J. Knight, of Connecticut; A. H. Stevens, of New York; J. Moultrie, of South Carolina; and A. H. Buchanan, of Tennessee.

*For Secretaries*—Drs. A. Stille, of Philadelphia; and J. R. W. Dunbar, of Baltimore.

*For Treasurer*—Dr. Isaac Hays, of Philadelphia.

A ballot was then had, and the persons named in the report were unanimously elected officers of the association for the ensuing year.

Dr. Chapman, President elect, was introduced, and on taking the chair made the following remarks:

He said he could find no language to express the depth of his gratitude. It had often been his good fortune during his professional life to have been complimented in the same manner, though not in the same degree. This was the most precious of all the honors he had received, as spontaneously conferred by his own brethren. He confessed his incompetency to serve the association as he could desire. He said he loved his profession, and should be ungrateful if he did not: whatever he possessed in this life had been bestowed by its favors; when he forgot it or deserted it and its disciples, he remarked with great emphasis, may God Almighty forget and desert me. He desired that the association should be persuaded of his ardent wishes for the cause, and that his most strenuous efforts would be unceasingly directed to advance the dignity of the profession, and extend its usefulness.

The following was offered by Dr. John B. Johnson, of Missouri:

Whereas, numberless and important evils result from the almost universal practice of allowing persons wholly ignorant of drugs and medicines to engage as apothecaries; and still greater from the universal traffic in patent and secret medicines; therefore,

*Resolved*, That the committee on education inquire into the expediency of establishing a school or schools of pharmacy in the respective States, for the special purpose of preparing persons for the business of apothecaries; and also the expediency of adopting a rule that no physician ought to patronize a druggist or apothecary who deals in patent and secret medicines, and report at the next annual meeting of the association. Adopted.

The following was offered by Dr. F. Campbell Stewart, of New York:

*Resolved*, That the committee on publication be directed to have published not less than two thousand copies of the proceedings of the late convention, with the reports, and of this association up to the time of its adjournment; and distribute the same to the members of the association. Passed.

After which, on motion, the association adjourned to meet in Baltimore on the *first Tuesday* of May, 1848.

For much of the foregoing account we are indebted to the Medical Examiner.

We hope soon to receive the official Journal of the proceedings of the convention, with the interesting reports acted upon, when our readers shall hear of it again.

